# Spin-crossover in metal complex-promoted homogenous catalysis

**DOI:** 10.3389/fchem.2026.1854732

**Published:** 2026-06-22

**Authors:** Lu Huang, Jean-Pierre Djukic

**Affiliations:** Laboratory of Organometallic Chemistry and Systemics, Institute of Chemistry UMR 7177/CNRS, Université de Strasbourg, Strasbourg, France

**Keywords:** coordination complexes, homogeneous catalysis, organometallic complexes, spin crossover, switchable catalysis

## Abstract

Spin crossover (SCO) is a well-established phenomenon by which transition-metal complexes can reversibly switch between low- and high-spin states, a phenomenon widely studied in materials chemistry. Recently, transition metal complexes that exhibit SCO have attracted increasing attention as catalysts in chemical reactions. Combining SCO with catalysis presents a promising strategy for creating switchable catalytic systems sensitive to light, pressure, and thermal stimuli. This review briefly covers the basics of SCO studies and the primary external stimuli that influence them. The following sections examine recent research on the role of SCO in homogeneous catalytic reactions with various transition-metal complexes and highlight its impact on reaction pathways, recommending the systematic evaluation of SCO’s effect on catalysis when investigating new 3*d* transition-metal-based catalysts.

## 1 Introduction

### 1.1 General considerations on spin crossover

Spin crossover (SCO) (Gütlich, 2013) refers to a reversible transition phenomenon between low-spin (LS) and high-spin (HS) electronic states in a transition metal center, typically triggered by external stimuli such as temperature (Bousseksou et al., 2004; Brooker, 2015; Moritomo et al., 2002), pressure (Gutlich et al., 2005; Levchenko et al., 2018; Li et al., 2021), light (Nakaya et al., 2021; Sciortino et al., 2014; Zhang et al., 2023), magnetic (Bousseksou et al., 2002) or even electric (Lefter et al., 2016; Utesov et al., 2019) fields. Most commonly studied in SCO research, be it in the solid state or in solution, are six-coordinate complexes of first-row transition metals with *d* ^4^-*d* ^7^ electron configurations.

**FIGURE 1:**
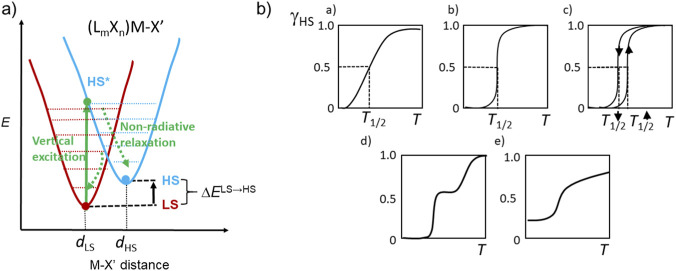
**(a)** Simplified diagram of the SCO mechanism for a prototypical (L_m_X_n_)MX’ complex, implying a vertical excitation of the LS state, a system LS-HS crossover, and the non-radiative relaxation to the HS state, resulting in a sensible change of the coordination geometry at the metal complex (changes of metal-ligand distances). Dashed lines correspond to molecular vibrational levels (Ekanayaka et al., 2022). **(b)** Schematic representation of the main thermoinduced SCO γ_HS_(*T*) curves (Gütlich and Goodwin, 2004).

From a physical point of view, SCO is a dynamic physical process that entails a variety of mechanisms that depend on the applied stimuli (Ekanayaka et al., 2022). A simplified representation of the SCO mechanism (Figure 1a) consists of considering a vertical excitation of an LS system of a typical (L_m_X_n_)M-X′ molecule and the transformation of the excited LS state into an excited HS state through a system crossover transition between two potential energy surfaces.

One of the major consequences of the LS→HS SCO transition is the dynamic change of the metal’s electron configuration that consequently dynamically affects the coordination geometry of the latter with its surrounding ligands, destabilizing some bonds while strengthening others. How the reactivity of the molecular system undergoing SCO is modified relates to the exceptional features in catalysis outlined in this survey.

### 1.2 Experimental methods of characterization of SCO

Here are briefly surveyed SCO characterization techniques in molecular systems relevant to potential use in solution catalysis, drawing on methodologies developed over decades in the field of molecular magnetism (Gütlich, 2013). As the SCO transition entails the spontaneous formation of paramagnetic species, almost all the methods used to characterize magnetism are *de facto* valid to establish SCO features, in the solid state, as well as in frozen and liquid solutions to some extent.

Nuclear magnetic resonance (NMR) is a particularly common spectroscopic technique in research laboratories today and a standard aid to synthesis. ^1^H NMR spectroscopy of liquid solutions provides a first diagnostic of the existence of SCO by producing temperature-dependent, broadened, and variably large frequency window spectra as a result of the effect of the paramagnetic character of HS states over the relaxation constants of proton nuclei. It is considered as a pertinent first tool for addressing SCO transitions in solution (Babailov et al., 2024). A well-established method for the estimation of the molecular magnetic susceptibility of a molecule by NMR spectroscopy is the one proposed by Evans (1959), which requires a minimal setup consisting of two coaxial NMR sample tubes, one filled with a solution of a mixture of the SCO-endowed analyte and a reference organic solute, the other containing a solution of about the same concentration of organic reference solute in the same solvent. The relative shift in the frequency of typical signals of the organic solute in contact with the SCO material is the variable of interest that allows determination of the magnetic susceptibility of the SCO material in solution at the NMR experiment’s temperature (Grant, 1995). By extension, *χ*_M_ *T*(*T*) curves can be drawn by undertaking variable temperature NMR experiments, which are directly comparable to those acquired by a SQUID magnetometer (see below).

Electron paramagnetic resonance (EPR) a.k.a. electron spin resonance (ESR) spectroscopy (Polash et al., 2023) is another method adapted to the characterization of HS states even though requiring generally the use of solid state samples to produce EPR spectra of acceptable resolution (Ju et al., 2024). While frozen solutions at subambient temperature may result in flat spectra due to poor population in the HS state, liquid solutions of an SCO-analyte generally produce insufficiently resolved spectra. EPR is a significant tools for analyzing the structure of HS spin states and complements valuably other techniques (Domracheva et al., 2013).

The use of superconducting quantum interference device (SQUID) magnetometry (Buchner et al., 2018; Foner, 1981) is generally recommended for characterizing SCO transitions in the solid as well as in solution giving access to reliable *χ*_*Μ*_ *Τ*(*T*) curves, allowing a clear diagnostic of SCO behavior.

While physically restrained to the analysis of solid (crystal) state samples to achieve the required recoilless *γ*-ray resonance absorption condition, Mössbauer spectroscopy (MöS) has witnessed developments (Burger and Vértes, 1983) to achieve exploitable liquid solution analysis by confinement into special porous glass capillary tubes (Bajnóczi et al., 2014), but has not yet achieved wide popularity. MöS is particularly important for iron (II) compounds because it can directly characterize spin states through hyperfine parameters, such as isomer shift and quadrupole splitting, of the iron center. An illustrative example (Kucheriv et al., 2016) is the complex [Fe(H*trz*)_2_(*trz*)](BF_4_) (Htrz = 1*H*-1,2,4-triazole), where SQUID analyzes revealed a clear spin transition temperature of 377 K upon heating and 344 K upon cooling, indicating the reversible conversion between the high-spin and low-spin states. Mössbauer spectroscopy showed a doublet corresponding to Fe (II) in the low-spin state (*δ* ^LS^ = 0.423 mm s^−1^ and quadrupole splitting *Δ*_EQ_^LS^ = 0.286 mm s^−1^), together with a small residual high-spin fraction.

The literature suggests that combining different techniques (Pavlov et al., 2020) is beneficial, even though, as shown below, it is rarely used in the investigation of SCO- catalysts. Rajamaran and Lescouezec et al. (De et al., 2018) have shown for a series of Fe(II) molecular complexes analyzed by SQUID and the Evans NMR method (complemented by the temperature monitoring of specific ^1^H signals) that transition temperatures *T*_1/2_ were consistent within 20 K in the solid state and solution, highlighting above all, the relevance of the Evans method as a “cheap” first analytical tool.

In addition, single crystal and powder X-ray diffraction techniques with or without external stimuli, are particularly instrumental to characterize the structural changes occurring upon SCO transitions (Guionneau, 2014).

### 1.3 Temperature-induced SCO

From a thermodynamic viewpoint, thermally induced SCO is promoted by the entropy gain at the HS state (Konig et al., 1985) resulting from electronic and vibrational contributions responsible for the system crossover (Nicolazzi and Bousseksou, 2018): wherever SCO is observed without other stimuli but temperature, it implies a relatively low energy excitation of an electron of the ground LS state producing an excited LS state that undergoes system crossover to an excited HS state, which, upon non radiative relaxation, yields a HS state. The change of the Boltzmann populations in LS and HS states allowed by SCO is a dynamic process, reversible when the two states show no reactivity, dependent on Δ*E* ^LS→HS^ energy gap (Figure 1a).

The thermally-induced spin transition is typically described by the molar fraction of high-spin species, γ_HS_(*T*), as a function of temperature. As shown in Figure 1b, depending on how γ_HS_ varies with temperature, spin transitions in solid materials and in solution to some extent can be categorized into several types: a) gradual transitions, b) abrupt transitions, c) spin transitions with hysteresis, d) two-step transitions, e) incomplete transitions (Gutlich et al., 2013).

An example was reported by Hiiuk and co-workers (Hiiuk et al., 2019), who synthesized a cyanoheterometallic coordination polymer with a bicyclic ligand 1,6-naphthyridine of composition [Fe(1,6-naphthy)_2_(Ag(CN)_2_)_2_]. The compound exhibits a 19 K-wide thermal hysteresis centered near RT (297 K): the crystal is in an LS state at room temperature, whereas it transitions to an HS state upon heating to 310 K. This spin transition is accompanied by a color change from red to orange. Furthermore, the average Fe-N bond length increases from 1.970(7) Å in the low-spin state to 2.143(10) Å in the high-spin state. Graf and co-workers (Graf et al., 2010) reported a first known low-spin cobalt(II) complex [Co(L-N_4_tBu_2_)(*dbsq*)](B(*p*-C_6_H_4_Cl)_4_) (L-N_4_ *t*Bu_2_ = dibutyl derivative of the tetraazamacrocyclic ligand 2,11-diaza[3,3](2,6)pyridinophane, *dbsq*- = 3,5-di-tert-butylsemiquinonate) which undergoes a spin transition from low-spin to high-spin state with increasing temperature rather than valence tautomerism. Structural analysis indicates that the bond lengths of the Co-N(py) and Co-O increases upon heating. Furthermore, SQUID magnetic measurements show that the χ_m_(T) value gradually increase from 200 K to 400 K, further confirming the progressive spin transition from the low-spin to the high-spin state.

### 1.4 Light-induced SCO

Light-induced SCO (Ekanayaka et al., 2022; Hauser, 2004) follows a similar path to the temperature induced one, but implies the relaxation of higher energy excited states, such as metal to ligand charge transfert (MLCT) ones, by various mechanisms that occur in the femto to the picosecond rate domains, the kinetics of which can be investigated by ultrashort laser pulse spectroscopy.

Two main effects can be instrumental in light-induced SCO experiments. The *light-induced excited spin-state trapping* (LIESST) (Hayami et al., 2000) acts directly on the metal center and typically occurs at low temperature, while the *ligand-driven light-induced SCO* (LD-LISC) (Boillot et al., 2004), implies the SCO transition to be indirectly regulated by light-induced changes in the ligand.

The initial example of LIESST was proposed by Decurtins and colleagues (Decurtins et al., 1984). They discovered that the iron(II) complex [Fe(*ptz*)_6_] (BF_4_)_2_ (where *ptz* = 1-propyltetrazole) facilitates spin conversion from the LS state to the HS state under irradiation with a 450 W xenon arc lamp. The spin state conversion is quantitative, and the trapped HS state remains stable without decay for several hours at temperatures below 50 K. The LIESST effect is a significant mechanism for achieving light-controlled spin transitions in SCO systems. However, its practical application is limited to low temperatures and to solid materials or frozen solutions.

**FIGURE 2:**
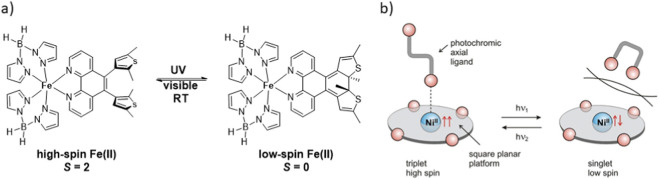
**(a)** Schematic representation of the spin-state switching of iron(II) complex entailing a reversible C-C bond creation. (Milek et al., 2013). **(b)** Schematic illustration of light-induced spin-state switching in square planar Ni(II) complexes through the association and dissociation of a photochromic axial ligand with a reversible C-C bond creation. (Thies et al., 2011). (Reprinted with permission, Copyright © 2011, American Chemical Society).

Rosner and colleagues (Milek et al., 2013) reported an example of LD-LISCI, in which they created a molecular iron(I) spin-crossover complex with a diarylethene ligand that can be photoisomerized, enabling efficient switching between the paramagnetic high-spin and diamagnetic low-spin states at room temperature in solution (Figure 2a). Repeated UV and visible light irradiation induces reversible isomerization of the ligand, which in turn triggers reversible SCO at the magnetic center. Under UV light, the spin-state conversion exceeds 40%, surpassing that of typical LIESST systems. Additionally, the photo-excited state remains thermally stable, with a half-life of about 18 days at room temperature in solution.

Herges’ group (Thies et al., 2011) introduced an innovative method to reversibly alter the magnetic properties of homogeneous solutions at ambient temperature through light-induced ligand-driven spin-state switching (LD-CISSS). Their approach utilized Ni-porphyrin as the foundational compound and azopyridines as photo-dissociable axial ligands. As depicted in Figure 2b, irradiation with visible light induces the ligand to assume a *trans* configuration characterized by minimal steric hindrance, thereby facilitating its coordination with the Ni(II) center to form a pentacoordinate complex. This coordination modification alters the metal’s electron configuration, resulting in a high-spin state. Conversely, ultraviolet light causes the ligand to adopt a cis configuration, which increases the steric hindrance and enables the Ni(II) metal to convert to a tetracoordinate state in a low-spin state. Quantitative verification via ^1^H NMR of the chemical shift of porphyrin pyrrole hydrogen signals demonstrated that light exposure can reversibly regulate the ratio of paramagnetic species in solutions at room temperature, reaching approximately 68%.

One year later, Thies et al. (2012) established the mathematical model and rational design principles of the LD-CISSS system. The results demonstrated that the maximum efficiency depends solely on the ratio of the association constants for both configurations. They further synthesized a new Ni-porphyrin with an improved photo-dissociable ligand. The results revealed that the association constant of the trans isomer is 5.36 times higher than that of the cis isomer. Correspondingly, efficient spin-state switching between the diamagnetic and paramagnetic states was achieved, with 72% paramagnetic Ni–porphyrin observed after irradiation at 365 nm and 32% paramagnetic species after irradiation at 440 nm.

### 1.5 Pressure-induced SCO

Applied to solid state samples of coordination complexes, pressure generally stabilizes low-spin configurations because of their shorter metal-ligand bonds and smaller molecular volumes (Gaspar et al., 2018). Pressure variation acts as an external stimulus that can modulate the spin states of Fe(II) complexes in the solid state for instance. Piquer and colleagues (Cristina and Fernande, 2003) studied two iron(II) complexes, {Fe[HC(3,5-(CH_3_)*pz*_3_)_2_]}I_2_ and {Fe[HC(3,5-(CH_3_)*pz*_3_)_2_]}(BF_4_)_2_. The first complex gradually transitions from a high-spin to a low-spin state as pressure increases. In contrast, the second remains high-spin from ambient pressure up to 78 kbar and only switches to low-spin between 78 and 94 kbar. In a dinuclear Fe(II) complex with the chemical formula [{Fe(*bpp*)(NCS)_2_}_2_(4,4′-*bipy*)]·2MeOH (*bpp* = 2,6-bis(pyrazol-3-yl) pyridine and 4,4-*bipy* = 4,4-bipyridine, DFT analysis reveals that the spin state of the iron(II) centers changes from a high-spin state (*S* = 2) to a low-spin state (*S* = 0) with increasing pressure (Chakraborty et al., 2020). Although the increased pressure shortens Fe-N bond lengths and induces angular distortion of the octahedral geometry, this iron complex can fully convert to the low-spin state without a complete structural phase transition.

## 2 SCO in material science

Since the first SCO complexes were reported by Cambi and Szegö (1931), an increasing number of SCO complexes have been synthesized and systematically studied. Fe(II) SCO compounds are the most thoroughly examined, due to the natural abundance of iron and its favorable electronic configuration, which allows reversible switching between a diamagnetic low-spin (*S* = 0, abbr. LS) state and a paramagnetic high-spin (*S* = 2, abbr. HS) state (Gutlich et al., 2013; Harding et al., 2016). Since the transition between LS and HS states is accompanied by changes in structure (molecular volume, metal-ligand bond lengths) and physicochemical properties, including variations in color, and magnetic moment of materials, the potential use of such materials as molecular switches, molecular actuators, memory storage devices, and other functional solid state molecular systems is actively investigated (Camarero and Coronado, 2009; Cornia and Seneor, 2017; Estrader et al., 2017; Gamez et al., 2009; Huang and Li, 2020; Kahn et al., 1992; Kahn and Martinez, 1998; Kumar and Ruben, 2021; Puebla et al., 2020; Rocha et al., 2005; Senthil Kumar and Ruben, 2017). Besides Fe(II) SCO complexes, SCO behavior has also been observed and increasingly investigated in other transition metal complexes, including Fe(III) (Harding et al., 2016; Pogany et al., 2026), Co(II) (Furmeyer et al., 2020; Palion-Gazda et al., 2017), Co(III) (King et al., 2012), Mn(III)(Ghosh et al., 2020; Wang et al., 2010), and Ni(II) (Brandenburg et al., 2018; Thies et al., 2010).

## 3 SCO in catalysis

### 3.1 The Two(Multi)State reactivity, a corner stone in SCO-promoted catalysis

The *two-state reactivity* (TSR) concept was initially proposed by Schröder, Shaik, and coworkers (Schröder et al., 2000) to describe how changes in the spin state during a reaction can influence the energy barrier of kinetically determining steps. As a consequence, when more than two spin states are involved, this is called *multistate reactivity* - or MSR - (Shaik, 2020). In this theoretical framework, a specific SCO transition occurs at the minimum-energy crossing point (MECP), which corresponds to the lowest-energy geometry at which two potential energy surfaces with different spin multiplicities overlap before reaching a key transition state. Although originally proposed for gas-phase reactions, like most theoretical models, the fundamental principles of T(M)SR are valid in homogenous solution and by way of consequence in heterogenous systems.

While several reports on transition metal catalysis outlined the contribution of spontaneous SCO occurring within the reaction’s course at various key steps of a catalysis (see below), the role of T(M)SR was not systematically assessed. Its main advantage is to highlight the fundamental notion of coexisting, spin-state defined reaction potential energy surfaces: SCO transitions–based mixing and intertwining of reaction potential energy surfaces contribute in this case to the minimization of the energetic payload required to achieve a given reaction.

**FIGURE 3:**
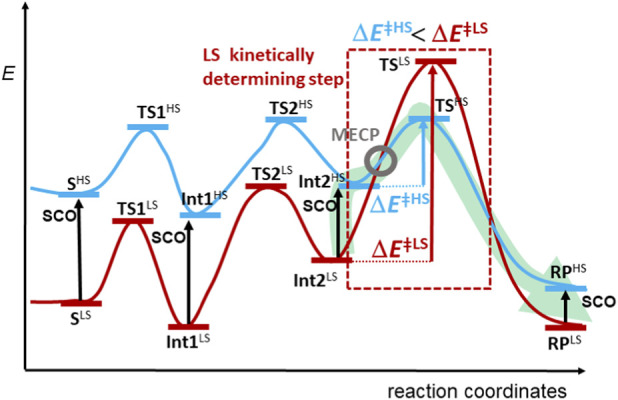
Idealized representation of the Two State Reactivity (TSR) reaction energy potential profiles complemented with SCO transitions for a reaction in which the low spin state (LS) and high spin state (HS) potential surfaces (here materialized by favored potential path curves colored in blue or red) cross at the MECP of a kinetically determining step (here, the conclusive step affording the final product RP upon the passing of transition state TS), where the HS “reaction mode” requires a lower activation barrier than the LS one (Δ*E* ^ǂHS^<Δ*E* ^ǂLS^). S stands for the metal-containing substrate, and Int for any reaction intermediate: for convenience, the structures of the LS and HS states are considered geometrically related. The bold green arrowed line materializes the preferred energy path around the kinetically determining step.

Considering that SCO may occur spontaneously in a coordination complex at which a catalytic transformation is unfolding, the population of the HS reaction potential energy surface is expected to be influenced by external stimuli (temperature, light exposure), compelling to consider both LS and HS states of the substrate and intermediates as crucial contributors of the reaction’s outcome (Figure 3).

In transition-metal-based catalysts, reactions can therefore be considered as potentially proceeding along either LS and HS states reaction potential energy surfaces. If no SCO occurs at substrates and intermediates, the reaction may proceed along the lowest potential energy surface (here the LS state), until the system reaches the MECP. At the latter point, the spin state crossover condition of the TSR is met: the two potential energy surfaces are degenerate and thus enable the SCO to a higher spin state yielding a lower activation energy pathway that facilitates the reaction.

Depending on the readiness of the metal-based system to achieve SCO transitions in the condition of the catalysis, as the reaction unrolls, the MECP phenomenon may also be complemented with trivial SCO transitions at reaction intermediates that contribute to populate the HS state reaction potential energy surface.

According to this general framework, any catalyst and its derivatives formed in the course of a catalytic transformation, endowed with SCO properties, may express combined SCO/T(M)SR features (Figure 3). The examples surveyed below illustrate this.

### 3.2 Iron-catalyzed systems

**FIGURE 4:**
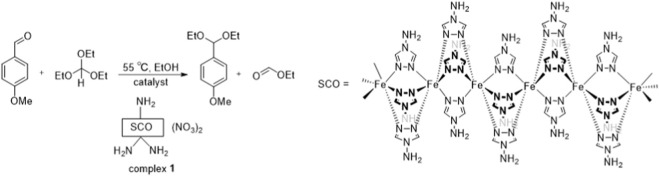
Reaction model selected to study the effect of spin state on the catalytic efficiency of complex 1 (Lai et al., 2025).

The use of iron complexes in spin-crossover catalysis has gained increasing recognition due to the close energy levels of their multiple spin states. These complexes can switch between low-spin (LS) and high-spin (HS) states, often leading to changes in their catalytic behavior. For instance, Gural’skiy and colleagues (Gural’skiy et al., 2017) investigated the Fe(II) coordination polymer [Fe(NH_2_ *trz*)_3_]Br_2_ (where NH_2_ *trz* is 4-amino-1,2,4-triazole) in the redox oxidation of 3,4,5,6-tetrachlorocatechol (TCC), using 3-chloroperoxybenzoic acid (CPBA) as the oxidant. They found that the low-spin state was more catalytically reactive than the high-spin state. The Mössbauer analysis also revealed that oxidation favored the formation of the low-spin complex. Likewise, Lai et al. (Lai et al., 2025) showed that shifting the iron spin state could notably influence catalytic efficiency in acetalization reactions (see Figure 4), with the high-spin form producing 1.7 times higher acetal yield than the low-spin form.

In catalytic reactions, SCO can lower the activation energy and facilitate transitions between potential energy surfaces, thereby altering the reaction pathway. For example, a high spin iron(II) complex, LFeEt (L = *β*-diketiminates), catalyzes β-hydride elimination. DFT studies show that the reaction involves a SCO from the HS to the LS state, thereby reducing the activation barrier and accelerating the process (Bellows et al., 2013).

**FIGURE 5:**
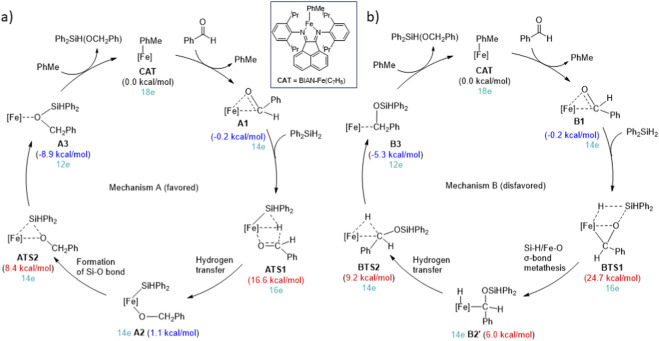
**(a)** Mechanism A: hydrogen transfer first followed by the formation of the Si-O bond. **(b)** Mechanism B: σ-bond metathesis of Si-H/Fe-O bond, first followed by the hydrogen transfer. The Gibbs free energies (in kcal/mol) of species in singlet, triplet, and quintet states are shown in black, red, and blue numbers, respectively (Fan et al., 2019).

Fan et al. (2019) studied the mechanism of the iron(0) complex BIAN-Fe(C_7_H_8_) (BIAN = 1,2-((bis-2,6-diisopropylphenyl)imino) and its catalyzed hydrosilylation of aldehydes, proposing two possible mechanisms (Figure 5). As shown, mechanism A is initiated by hydrogen transfer from silane to benzaldehyde, while mechanism B begins via *σ*-bond metathesis between Si-H and Fe-O bonds. DFT calculations and experimental results reveal that mechanism A is the favored pathway. The key step in mechanism A is the ligand-to-ligand hydrogen transfer process, with an activation energy of 16.8 kcal/mol. In contrast, the Gibbs free energy barrier for the σ-bond metathesis step in mechanism B is 24.9 kcal/mol, which is higher than in mechanism A. In mechanism A, SCO occurs between the triplet and quintet potential energy surfaces, lowering the activation energy and facilitating the reaction.

**FIGURE 6:**
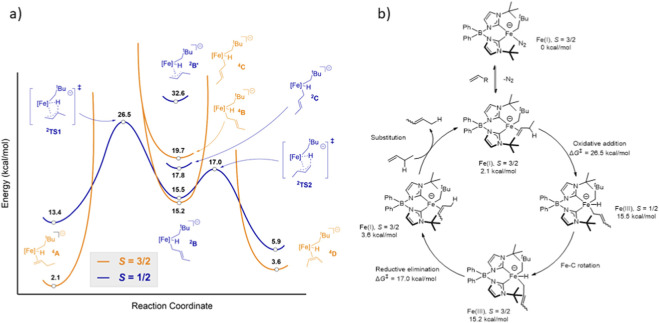
**(a)** Calculated reaction coordinate for alkene isomerization. Relative energies in kcal/mol. Orange and blue curves represent *S* = 3/2 and *S* = 1/2 potential energy surfaces, respectively (Reprinted with permission, Copyright © 2020, American Chemical Society). **(b)** The lowest energy computed catalytic cycle (Lutz et al., 2020).

Lutz et al. (2020) found that organometallic iron(I) complex [K(2.2.2-cryptand)][Ph_2_B(^t^Bu*Im*)_2_FeCH_2_^t^Bu(N_2_)] catalyzes alkene isomerization via the allyl mechanism. As shown in Figure 6a, the authors observed that the reaction occurs between two different spin states, exhibiting two-state reactivity (Figure 6a). Alkenes initially coordinate in the high-spin *S* = 3/2 state, but the key oxidative addition step, involving allylic C-H activation, can only happen in the low-spin *S* = 1/2 state (Figure 6b). In the high-spin state, the reaction is ‘spin-blocked’ because the metal orbital is occupied. Energy calculations show that the energy barrier for the oxidative addition transition state is much lower on the *S* = 1/2 surface. Therefore, the reaction proceeds via a spin-crossing to the low-spin state, completing the essential bond-forming and bond-breaking steps, producing isomerized products before returning to the high-spin state.

**FIGURE 7:**
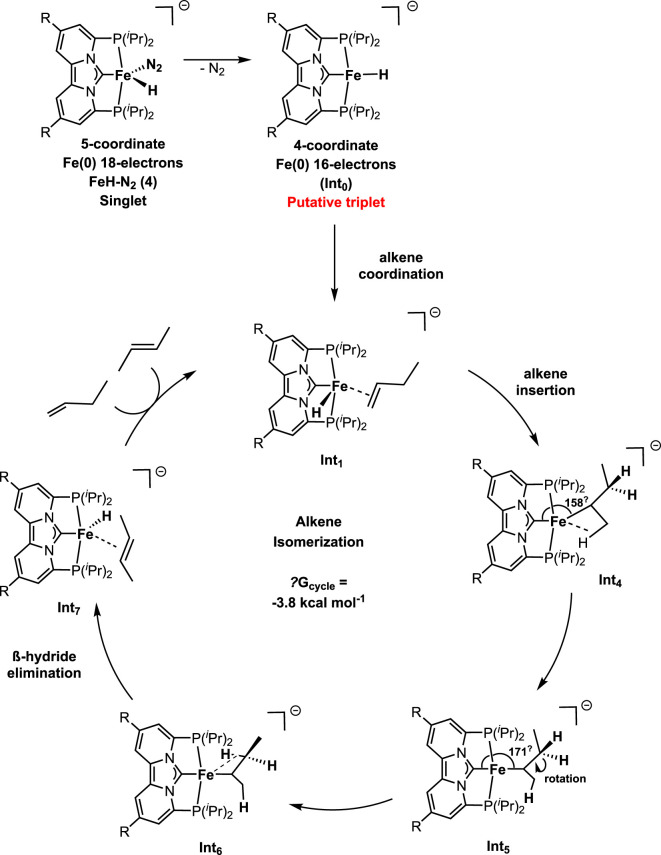
Plausible mechanism for alkene isomerization (to alleviate computational complexity, calculations were performed with a truncated model of the catalyst, R = Me) (Garhwal et al., 2021).

Garhwal et al. (2021) described an anionic iron hydride complex [(PCNHCP)Fe(H)N_2_]^-^ (where PCNHCP is a bi(diisopropylphosphino) N-heterocyclic carbene pincer ligand) that effectively catalyzes the isomerization of 1-alkenes to 2-alkenes with high reactivity and selectivity at room temperature. Mechanistic and DFT studies suggest that this reaction proceeds via an alkyl mechanism involving alkene insertion, β-hydride elimination, with high activity occurring on the triplet surface (Figure 7). Although the iron complex is in a singlet state at its ground state, it was speculated, based on computational studies in the absence of experimental proofs, that it probably transitions to a triplet state through SCO after N_2_ dissociation. This SCO allegedly lowers the energy barriers for key steps such as nitrogen gas dissociation, alkene insertion, and *β*-H elimination. The catalytic system achieves a turnover number (TON) of 160,000 and a turnover frequency (TOF) of 6600 h^-1^ at room temperature, outperforming many other precious metal catalysts.

**FIGURE 8:**
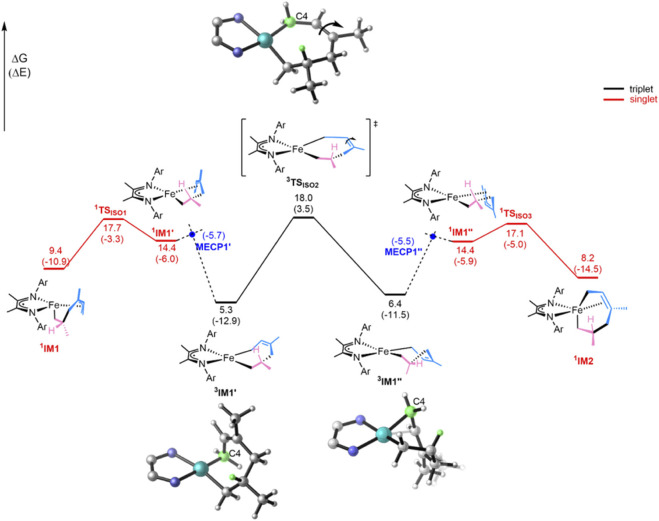
Isomerization energy profile. The relative free energies and electronic energies (in parentheses) are given in kcal/mol. The central part in black (triplet state) is the main process flipping the coordinating face of the diene moiety (Zhang et al., 2020) (Reprinted with permission, Copyright © 2020, American Chemical Society).

Zhang et al. (2020) investigated the mechanism of 1,4-selective hydrovinylation of 1,3-dienes catalyzed by iron-diimine ligands via DFT calculations. The results revealed that the reaction does not proceed along a single spin potential energy surface but involves multiple spin-crossover events between the triplet and singlet states, exhibiting two-state reactivity (Figure 8). The triplet state is more favorable for stabilizing intermediates, while the singlet state is more favorable for the key bond-forming steps, oxidative coupling, and *β*-hydrogen migration. The alleged reason is that low-spin Fe(III) has more vacant *d* orbitals, which enable stronger metal-substrate orbital interactions, thereby lowering the bond-formation energy barrier.

**FIGURE 9:**
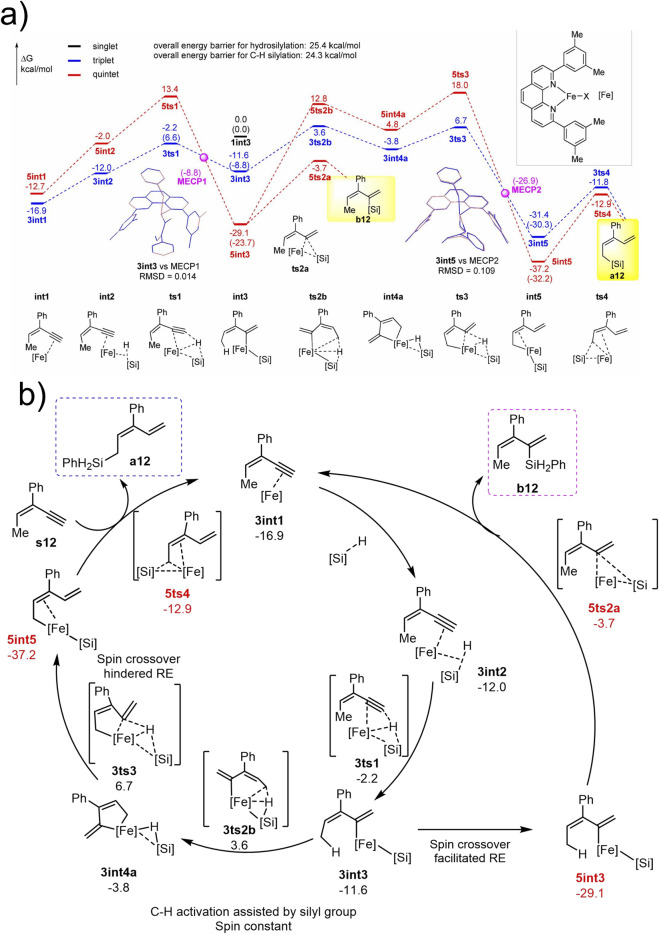
**(a)** Calculated energy profile (Reprinted with permission, © 2024 Wiley–VCH GmbH ); **(b)** proposed catalytic cycle (He et al., 2024a).

In a catalytic system, SCO influences both the chemical selectivity of the reaction and the rates of individual steps, which can either accelerate or inhibit reaction. For instance, He and colleagues (He et al., 2024a) investigated allylic C(*sp* ^3^)-H silylation and hydrosilylation of 1,3-enynes using open-shell 1,10-phenanthroline/iron complexes as catalysts. Their combined experimental and DFT studies revealed that SCO enables a single intermediate to yield different products by accessing distinct spin-state potential energy surfaces. As shown in Figure 9a, the reaction mainly proceeds on the triplet surface, where Si-H transfer to the alkyne forms the common intermediate **3int3**. The system can stay on the triplet surface for allylic C(*sp* ^3^)-H activation or switch to a quintet state via triplet-to-quintet crossing at MECP1, forming a more stable quintet intermediate **5int3**, which is a typical feature of two-state reactivity. This then undergoes reductive elimination to give a hydrosilylation product **b12**, or continues on the triplet surface through C-H activation and hydride migration to produce an allylic C(*sp* ^3^)-H silylation product **a12** (Figure 9b). Additionally, they showed that SCO can have an opposite effect during later stages of the reaction, where it shifts the system into a HS state with higher reductive elimination barriers, thus slowing the reaction.

In the same year, He et al. (2024b) revealed that the iron-catalyzed hydrosilylation of alkynes is a typical spin-crossover catalysis. Both experimental and DFT calculations indicate that the reaction involves two potential energy surfaces: triplet and quintet. Oxidative addition predominantly occurs on the triplet potential energy surface, whereas reductive elimination is more favorable on the quintet surface. SCO reduces the overall reaction energy barrier by approximately 8.4 kcal/mol, thereby significantly accelerating the reaction rate.

### 3.3 Cobalt-catalyzed systems

Although iron compounds have been studied more extensively in SCO catalysis, a growing body of literature indicates that SCO in cobalt complexes can also affect catalytic activity. SCO can effectively lower the energy barriers at key steps, thereby promoting the reaction’s progression. Yu et al. (2019) investigated the mechanism of the Cp*Co(III)-catalyzed amination of non-activated C(*sp* ^3^)-H bonds through systematic theoretical studies. The results indicated that the reaction does not proceed on a single spin-state potential-energy surface but instead exhibits a distinct two-spin-state reactivity scenario. During the C(*sp* ^3^)-H activation step, SCO at the MECP lowers the reaction energy barrier and promotes reaction progress.

**FIGURE 10:**
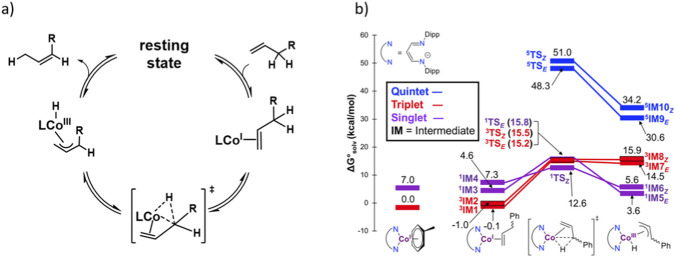
**(a)** Proposed allyl mechanism; **(b)** Calculated free energies of intermediates (IM) and transition states (TS) during alkene isomerization (Kim et al., 2021). (Reprinted with permission, Copyright © 2021, American Chemical Society).

Kim and co-workers (Kim et al., 2021) reported a highly spin-excitable Co(I) β-diimide complex capable of highly *Z*-selective double bond migration in simple alkenes and allylic aromatics. This system enables the selective formation of *Z*-2-alkenes under kinetic control (*Z/E* up to 94:6) and demonstrates outstanding performance even with allylbenzene-type substrates. Mechanistic studies indicate the reaction proceeds via a π-allyl mechanism (Figure 10a). The calculated free-energy diagram shows that the catalyst exists in a triplet ground state, while the lowest-energy oxidation-addition transition state is on the singlet potential energy surface. This suggests that SCO occurs at a key reaction step, lowering the energy barrier. More importantly, on the singlet potential energy surface, the transition state leading to the *Z*-product is significantly lower than that leading to the E-product (Figure 10b). Therefore, the SCO not only reduces the overall activation energy but also improves stereoselectivity, allowing the catalyst to favor the thermodynamically less stable *Z*-alkene.

**FIGURE 11:**
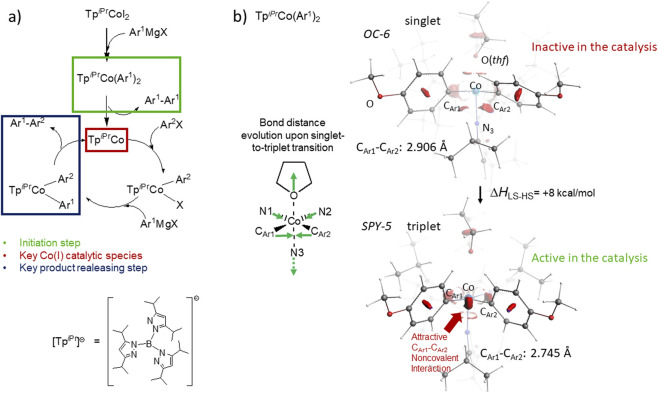
The Corriu-Kumada reaction catalyzed by a Co(III) trispyrazolylborate complex: **(a)** the simplified catalytic cycle outlining the catalyst initiation step, leading to the spin state-sensitive formation of the catalytic Co(I) species, followed after an oxidative addition step by the spin-state sensitive reductive elimination step, **(b)** the distinct noncovalent interaction (NCI) signatures of the Co(III)(Ar^1^)_2_ intermediate in geometrically distinct *OC*-6 singlet and *SPY*-5 triplet spin states as computed by the DFT, rationalizing their higher propensity of undergo the reductive elimination of Ar^1^-Ar^1^ when at the triplet spin state (Wang et al., 2024).

Djukic’s group (Wang et al., 2024) reported that the Kumada–Corriu hetero-coupling of haloarenes with arylmagnesium bromides can be efficiently catalyzed (>80% yield) by the new cobalt complex Tp^iPr^CoI_2._ The reaction is significantly enhanced under light irradiation compared to darkness. The Evans method (Evans, 1959) established the SCO behavior of the key Co(III) species in the catalysis. Mechanistic studies have shown that the catalytic activity is controlled by the coexistence of low-spin and high-spin states (approximately 3:2 ratio) in the key intermediate Tp^iPr^CoAr_2._ The active intermediate is the triplet one, which can also generate the key triplet Co(I) species (Figure 11). DFT calculations show that the reductive elimination activation barrier for the triplet state of the biaryl-Co(III) intermediate is significantly lower than that for the corresponding singlet state, making the HS state a catalysis enabler. This energetic propensity of the HS state, which holds different geometric properties as compared to the LS (*d*(C_Ar1_-C_Ar2_ is lower in the HS state than in the LS state), has been correlated to the buildup of an attractive non-covalent interaction (NCI) domain in the prereactive triplet state complex Tp^iPr^Co(Ar^1^)_2_ species in the segment separating C_Ar1_ from C_Ar2_, which is totally absent in the LS counterpart. This attractive NCI buildup (Cornaton and Djukic, 2021; Figueirêdo de Alcântara Morais et al., 2026b) illustrates the propensity of the HS state to undergo an easier C-C coupling between C_Ar1_ and C_Ar2_ (Figure 11). This system also demonstrates how SCO and external stimuli such as light irradiation can dynamically affect reaction pathways and enable catalysis without the need of an added photosensitizer.

**FIGURE 12:**
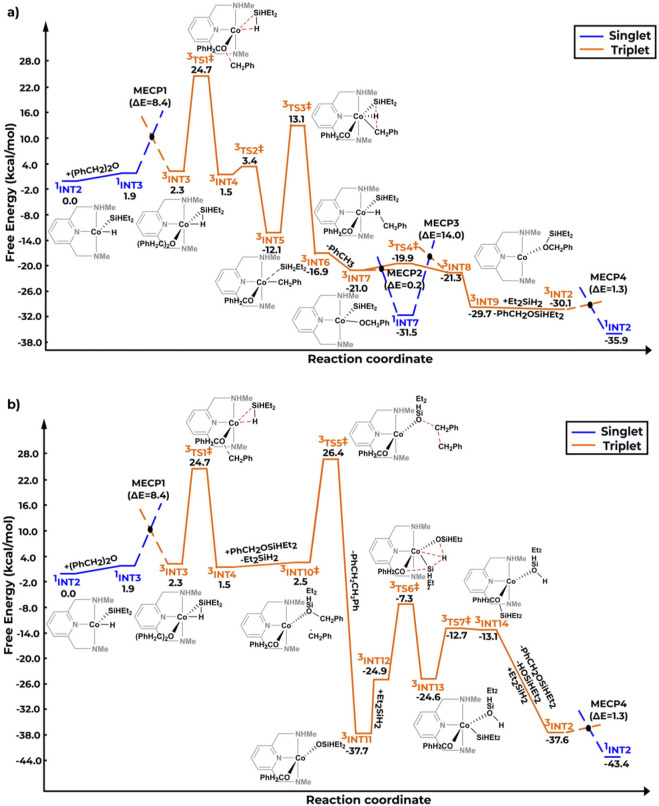
Free energy landscape computed with implicit solvation for toluene: **(a)** C-H coupling mechanism and **(b)** C-C coupling mechanism. All energy values are in kcal/mol, relative to those of 1INT2 taken as zero. (Sahu et al., 2026). (Reprinted with permission, © 2025 Wiley-VCH GmbH).

Recent studies (Sahu et al., 2026) have identified a cobalt pincer complex that can catalyze the conversion of both symmetric and asymmetric arylmethyl ethers, as well as aryl-alkyl ethers, into alkane products. DFT calculations show that once cobalt compounds generate an active Co-H species, this species must undergo a spin crossing from singlet to triplet at the MECP when coordinated with an ether ligand. This step facilitates C-O bond cleavage and the formation of a Co(II)-benzyl radical intermediate. The spin transition is essential for initiating the radical pathway. On the triplet energy surface, the radical can either abstract hydrogen to produce a C-H coupling or attack an aryl silyl ether to form a C-C coupling. Finally, the radical returns to the ground state through another spin crossing, completing the catalytic cycle (Figure 12).

### 3.4 Other transition metals

Compared to iron- and cobalt-based systems, only a limited number of studies on other 3*d* transition metals have been reported. The main reason is that the ligand-field splitting energy (Δ_oct_) is comparable to the electron-pairing energy (P) in iron and cobalt complexes, allowing both high-spin (HS) and low-spin (LS) states to be thermally accessible (Halcrow, 2016; Nicolazzi and Bousseksou, 2018). However, in other 3*d* transition metals, the difference between Δ_oct_ and P is either much smaller or larger, which favors a single spin state and thereby inhibits the SCO behavior. Furthermore, iron and cobalt centers are highly sensitive to changes in ligand-field strength and external stimuli, thereby enhancing spin-state switching properties. In transition metal compounds containing Ni, Mn, Ir and V SCO plays a key role in catalytic reactions.

**FIGURE 13:**
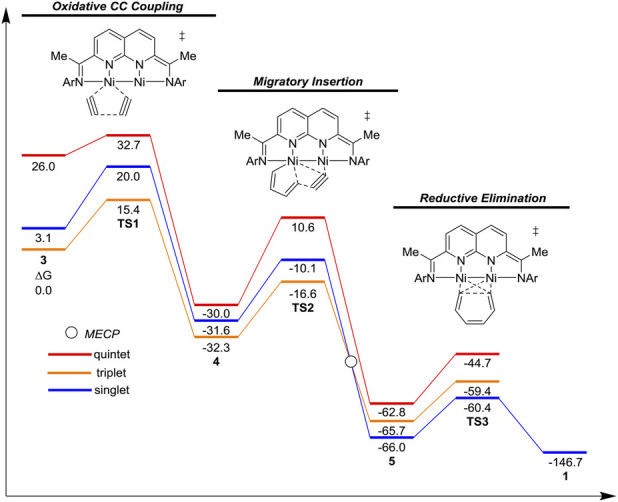
Outline of singlet, triplet, and quintet free energy surfaces and spin crossings for acetylene cyclotrimerization (Kwon et al., 2017). (Reprinted with permission, Copyright © 2017, American Chemical Society).

Kwon et al. (2017) reported that a dinuclear Ni complex catalyzes selective cyclotrimerization of monosubstituted alkynes. DFT calculations revealed that the reaction follows a SCO mechanism. After alkyne coordination, the system transitions from singlet to triple states. Oxidative C-C coupling occurs via **TS1** to form metallacyclopentadiene. Following migration insertion (**TS2**) also occurs at the lowest energy barrier on the triplet potential surface, forming a bridged metallacycloheptatriene intermediate (Figure 13). Since singlet and triplet energies are close, the system undergoes spin crossing at this point and proceeds via a low-energy barrier reduction elimination on the singlet potential surface to form the corresponding product.

**FIGURE 14:**
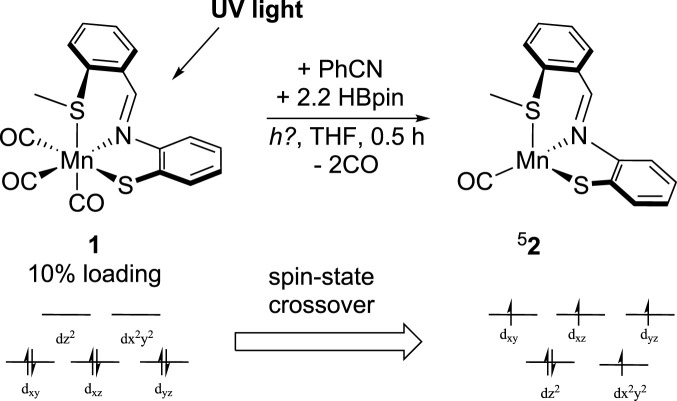
The SCO of catalysis under UV light (Elsby et al., 2022).

Elsby et al. (2022) studied the dihydroboration of nitriles with pinacolborane (HB*pin*) catalyzed by the Mn(I) complexes, Mn(*ĸ* ^3^-SMeNS)(CO)_3_. The authors found that the catalysis process requires the presence of UV light to sustain the reaction proceeds. Under UV light, CO dissociation generates a coordinatively unsaturated HS Mn species, which is the key step to proceed the catalytic cycles (Figure 14). EPR studies combined with DFT calculations indicated that the Mn center undergoes a spin-state change and preferentially adopts the high-spin configuration during catalysis, significantly lowering the energy barriers for key bond-activation steps. In the absence of SCO, the reaction becomes energetically unfavorable.

**FIGURE 15:**
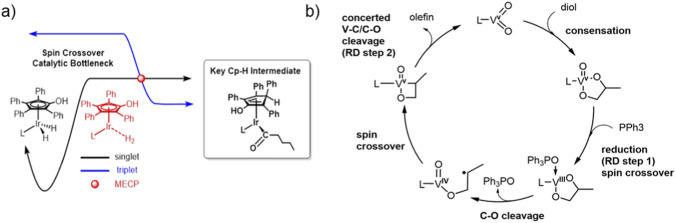
**(a)** 2D representation of the singlet (black colored line) and triplet (blue colored line) spin state surface crossing at the MECP (red colored structure) (Kirkland et al., 2023). (Reprinted with permission, Copyright © 2023, American Chemical Society) **(b)** Proposed Mechanism for V-Catalyzed DODH (Jiang et al., 2016).

SCO is also the key step determining the catalytic rate. Kirkland et al. (2023) systematically analyzed reaction pathway for aldehyde retro-hydroformylation catalyzed by cyclopentadienyl phosphine-type Ir(III) catalysts through DFT calculations and metadynamics simulations. They revealed that in the catalytic cycle, H_2_ reduction elimination step does not proceed via a conventional single-surface transition state, while the process requires a transition from the singlet to triplet at the minimum energy crossing point (MECP) (Figure 15a). This spin-state crossing reduces the energy required for H_2_ departure. Importantly, this MECP represents the highest energy bottleneck in the early stages of the cycle, thereby determining the overall rate of the catalytic reaction.

Jiang et al. (2016) studied the mechanism of vanadium-catalyzed deoxydehydration (DODH) of diols using DFT methods. The results indicate that this process involves two SCO events (Figure 15b). They demonstrated that the preferred mechanism begins with the condensation of vanadium with vicinal diol to form vanadium(V) diolate, which is further reduced by PPh_3_ to form the vanadium(III) diolate via the first SCO. The second SCO occurs after a single C-O cleavage to form the alkylvanadium(V) intermediate and is followed by a concerted V−O/C−O bond cleavage to generate the olefin product (Jiang et al., 2016). Although the main rate-determining step is reduction of the vanadium(V) diolate by PPh_3_, olefin release also influences the reaction rate to some extent. In this catalytic cycle, SCO not only facilitates the transformation between vanadium(III) and vanadium(V) species, but also promotes olefin release that enhances the reaction rate.

### 3.5 Electro and photo catalysis in solution

In addition to homogeneous catalysis, SCO has been extensively used in functional materials, and particularly in electrocatalysis and photocatalysis. For the sake of conciseness, this topic will be only briefly mentioned here, as it falls outside the subject of this survey.

In electrocatalysis, Liu et al. (2024) studied the spin effects of bimetallic MOFs (NiAl and NiFe) in the oxygen evolution reaction (abbr. ER). Computational results showed that SCO can occur in key intermediates under different potentials, thereby significantly affecting reaction free energies and activation barriers. Notably, the NiFe system improves electrical conductivity via Fe addition and adjusts the thermodynamics and kinetics of OER by controlling the spin state.

In photocatalysis, a {Fe-Pt} Hofmann-type coordination polymer with adjustable spin states enables Fe(II) to switch reversibly between high- and low-spin states, thereby controlling H_2_O_2_ production. The low-spin state promotes charge transfer between metals and lowers energy barriers for the oxygen reduction (ORR) and water oxidation (WOR) reactions (Huang et al., 2023).

## 4 Conclusion

Applications of SCO are spreading out from a focus in materials science to an emerging area in catalysis research. As understanding of multistate reactivity in transition-metal-mediated processes grows, SCO is progressively seen as a key factor influencing catalytic behavior. This review first examines the factors that induce SCO, then highlights recent advances in spin-crossover-regulated homogeneous catalysis. Recent studies indicate that in these systems, spin-state changes lower activation energy barriers at rate-determining steps, stabilize reactive intermediates, thereby impacting product formation efficiency and selectivity.

While it may be expected that SCO-catalysis will soon contribute to the field of “switchable catalysis”, the primary focus of stimuli-activated SCO-based catalysts still requires comprehensive studies to understand how to achieve the proper tuning of the LS-HS energy gap through the structural adjustment of the metal’s ligands. This endeavor requires the rationalization of the structural factors that affect significantly the SCO transitions relevant to the kinetically determining steps, which remains a rarely addressed question in the surveyed literature. Complementary to this endeavor is the rationalization of the change of reactivity of the metal’s ligands upon SCO transitions, as there exist, to the best of our knowledge, no theoretically grounded explanation of the significant changes of reactivity of the key atomic centers occurring upon a SCO transition. One possible direction to investigate is the electronic density redistribution occurring in isolated steps, which is best carried out with an analytical topological approach such as the Independent Gradient Model (Figueirêdo de Alcântara Morais et al., 2026a; Figueirêdo de Alcântara Morais et al., 2026b).

Switchable catalysis has quickly developed into a promising approach in sustainable and green chemistry, driven by the need for multifunctional catalytic systems that can adjust their reactivity and selectivity in response to external stimuli (Ghorbani-Choghamarani and Taherinia, 2022b). Incorporating SCO offers a promising, relatively unexplored route, to the development of switchable catalysts.

It is now evident that any development of novel organometallic applications of first-row 3*d* transition-metal complexes should evaluate whether SCO might be playing a role by opening channels of reactivity with lower activation barriers: the existence of multistate reaction mechanisms may consist of condition-dependent, intricately structured reaction energy profiles that contain numerous SCO and potential-energy curve crosspoints. In other words, what may appear as a fully LS–state mechanism may well hide one or more HS-state key steps, the evidence of which may contribute to an improved engineering of catalysts.

There is an imperious need to investigate systematically SCO-based systems from the view point of their physical properties. It appeared, while surveying the literature, the efforts to physically characterize SCO-endowed catalysts by *ad hoc* methods were often very limited. One major reason for this is the dynamic nature of the spin transition making SCO-endowed intermediates challenging to observe *in situ* or to isolate, constraining most studies to an intensive use of DFT modeling.

Finally, if most of the studies surveyed therein essentially focused on the catalytic performance of 3*d*-transition metal complexes, the fate of these complexes and their recyclability have yet to be addressed (Fassbach et al., 2024), which is widely overlooked. Unlike redox or photo-redox catalysis, SCO does not need auxiliary co-catalysts for activation. From this perspective, SCO-promoted catalysis has the potential to lead the way in the development of sustainable, industrial-scale homogeneous switchable catalysis (Ghorbani-Choghamarani and Taherinia, 2022a), as it potentially limits waste and should help meet industrial regulations on final product metal content (Armstrong et al., 2021; Azzopardi et al., 2023).
